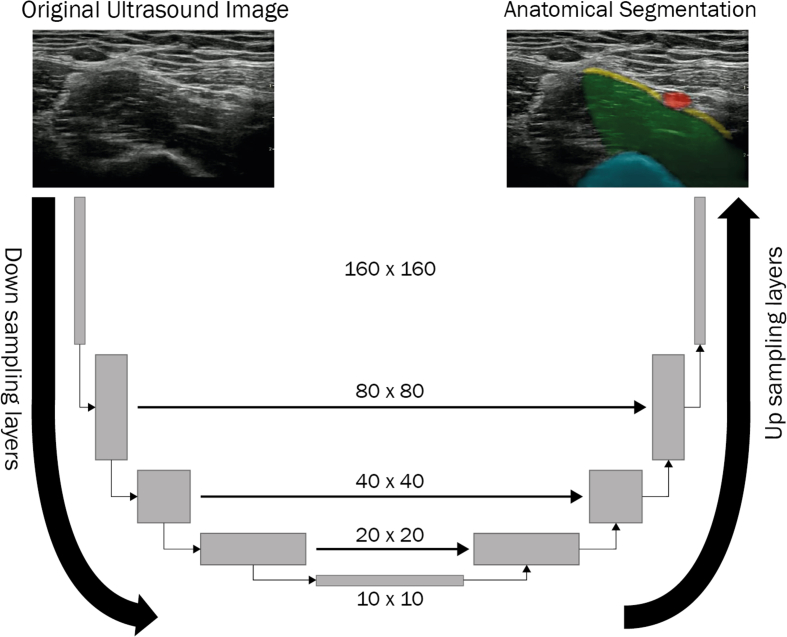# Artificial intelligence for ultrasound scanning in regional anaesthesia: a scoping review of the evidence from multiple disciplines

**DOI:** 10.1016/j.bja.2024.01.036

**Published:** 2024-03-05

**Authors:** James S. Bowness, David Metcalfe, Kariem El-Boghdadly, Neal Thurley, Megan Morecroft, Thomas Hartley, Joanna Krawczyk, J. Alison Noble, Helen Higham

**Affiliations:** 1 Nuffield Department of Clinical Neurosciences, University of Oxford, Oxford, UK; 2 Department of Anaesthesia, Aneurin Bevan University Health Board, Newport, UK; 3 Nuffield Department of Orthopaedics, Rheumatology & Musculoskeletal Sciences, University of Oxford, Oxford, UK; 4 Emergency Medicine Research in Oxford (EMROx), Oxford University Hospitals NHS Foundation Trust, Oxford, UK; 5 Department of Anaesthesia and Peri-operative Medicine, Guy's & St Thomas's NHS Foundation Trust, London, UK; 6 Centre for Human and Applied Physiological Sciences, King's College London, London, UK; 7 Bodleian Health Care Libraries, University of Oxford, Oxford, UK; 8 Faculty of Medicine, Health & Life Sciences, University of Swansea, Swansea, UK; 9 Intelligent Ultrasound, Cardiff, UK; 10 Institute of Biomedical Engineering, University of Oxford, Oxford, UK; 11 Nuffield Department of Anaesthesia, Oxford University Hospitals NHS Foundation Trust, Oxford, UK

**Keywords:** artificial intelligence, evaluation, medical devices, regional anaesthesia, regulation, standardisation, ultrasound

## Abstract

**Background:**

Artificial intelligence (AI) for ultrasound scanning in regional anaesthesia is a rapidly developing interdisciplinary field. There is a risk that work could be undertaken in parallel by different elements of the community but with a lack of knowledge transfer between disciplines, leading to repetition and diverging methodologies. This scoping review aimed to identify and map the available literature on the accuracy and utility of AI systems for ultrasound scanning in regional anaesthesia.

**Methods:**

A literature search was conducted using Medline, Embase, CINAHL, IEEE Xplore, and ACM Digital Library. Clinical trial registries, a registry of doctoral theses, regulatory authority databases, and websites of learned societies in the field were searched. Online commercial sources were also reviewed.

**Results:**

In total, 13,014 sources were identified; 116 were included for full-text review. A marked change in AI techniques was noted in 2016–17, from which point on the predominant technique used was deep learning. Methods of evaluating accuracy are variable, meaning it is impossible to compare the performance of one model with another. Evaluations of utility are more comparable, but predominantly gained from the simulation setting with limited clinical data on efficacy or safety. Study methodology and reporting lack standardisation.

**Conclusions:**

There is a lack of structure to the evaluation of accuracy and utility of AI for ultrasound scanning in regional anaesthesia, which hinders rigorous appraisal and clinical uptake. A framework for consistent evaluation is needed to inform model evaluation, allow comparison between approaches/models, and facilitate appropriate clinical adoption.

**Editor's key points**

• Artificial intelligence (AI) for ultrasound scanning in regional anaesthesia crosses multiple disciplines, which risks limited knowledge transfer between specialty silos and unnecessary duplication of work.
• This scoping review summarises the available evidence regarding the accuracy and utility of such technology. The literature shows that a standardised methodology for evaluation is needed, which can be adopted for academic, clinical, and regulatory purposes.
• A structured and consistent approach, with cross-disciplinary collaboration, may enable a full understanding of AI systems in regional anaesthesia and inform clinical uptake.

Ultrasound imaging for regional anaesthesia, first described in 1989,^1^ is the principal method to guide the targeted delivery of local anaesthetic during peripheral nerve blockade (PNB).^2^^,^^3^ Abdallah and colleagues^4^ described its use as ‘the most important advance in regional anaesthesia practice of the new millennium’ as it has been shown to enhance both efficacy and safety^3^ through visualisation of the needle tip and key sono-anatomical structures. It is also used to image the spine and surrounding tissues ahead of performing central neuraxial blockade (CNB; spinal and epidural).^5^ As a dynamic imaging modality, the quality of the views obtained depends on the skill of the operator and the image generated by the machine.^4^ Whilst ultrasound machines have advanced considerably since the inception of ultrasound-guided regional anaesthesia (UGRA), it is unclear whether clinicians' knowledge and skill in utilising this technology has progressed to a similar extent.^6^

Artificial intelligence (AI) is a field of computer science which uses techniques that enable computers to undertake tasks associated with human intelligence.^6^ Recent publications present the case for AI to aid in the acquisition and interpretation of optimal ultrasound images for UGRA (PNB and CNB).^6^^,^^7^ However, this is an interdisciplinary field that draws on contributions from multiple areas, including computer science, engineering, and clinical medicine. As a consequence, evaluation of AI technologies for the identification of anatomical structures on ultrasound in UGRA can be heterogeneous. For example, proof-of-concept studies in preclinical experimental work^7^^,^^8^ typically involve different approaches to early clinically-orientated assessments of commercially-available systems.^9^, ^10^, ^11^, ^12^

Lack of knowledge transfer between disciplinary silos risks duplication of work and hindering the development, evaluation, and adoption of this technology. It is therefore important to synthesise the available literature into a single coherent summary, which identifies areas of strength and deficiency in current evaluation approaches to inform further work in this nascent and rapidly evolving field. This scoping review aims to identify and map the available evidence on the accuracy and utility of AI systems for anatomical structure identification on ultrasound in regional anaesthesia.

## Methods

The protocol presented in this manuscript was developed using the guidance provided by the Joanna Briggs Institute (JBI) and Preferred Reporting Items for Systematic Reviews and Meta-Analyses—Extension for Scoping Reviews.^13^, ^14^, ^15^

### Prior scoping reviews and registration

When checked at the commencement of this study, on May 4, 2022, no scoping or systematic reviews were registered with the JBI Database of Systematic Reviews and Implementation Reports, Cochrane Database of Systematic Reviews, or the International Prospective Register of Systematic Reviews (PROSPERO).

The objectives, inclusion criteria, and methods for this scoping review were specified in advance and documented in a protocol registered on the medRxiv preprint server for health sciences before data extraction from the identified sources.^16^

### Scoping review question

The question addressed by this scoping review is, ‘*what is the evidence* supporting *the use of artificial intelligence for ultrasound scanning in regional anaesthesia?*’ As per published guidance,^13^^,^^14^ it is based on the PCC model, containing ‘population’ [human ultrasound scans], ‘concept’ [AI], and ‘context’ [UGRA] elements.

### Inclusion criteria

#### Population

Sources were included if they pertained to human ultrasound scanning (living or cadaveric studies). Data from animal and bench studies were excluded.

#### Concept

Sources included relate to the accuracy of AI-based anatomical structure identification on real-time B-mode ultrasound and the utility of these systems with respect to clinical practice. For the purposes of this review, a broad definition of utility was adopted and may refer to anaesthetists' ultrasound scanning performance, patient outcomes (PNB efficacy or safety), delivery, cost-effectiveness of URGA, or both.

#### Context

Sources relating to ultrasound scanning in the context of CNB (e.g. spinal and epidural) and PNBs were included.

### Types of sources

All publicly available data (including academic publications, clinical/specialist society information, and commercial product literature) were considered. For sources published in languages other than English, investigators contacted the author to request a translated version.

### Search strategy

#### Published academic literature

The search strategy was developed by the lead investigator (JSB), reviewed/modified by the author team, and then executed by a medical librarian (NT) in conjunction with the lead investigator. The strategy was explicitly designed to consider sources spanning computer science, engineering, and clinical medicine, to capture the multidisciplinary contributions to this field.

Five databases were searched from inception to April 14, 2023; Medline (OvidSP; 1946 – present), Embase (OvidSP; 1974–present), CINAHL (EBSCO; 1981–present), IEEE Xplore (IEEE: 1988–present), ACM Digital Library (ACM; 1951–present). An original search was run on Medline and Embase only on March 3, 2023. This was superseded by the searches on April 14, 2023. The search was comprised of title/abstract keywords and subject headings for AI, ultrasonography, and anaesthesia. No date or language limits were applied. References were exported to EndNote 20 (Clarivate, London, UK) for de-duplication. The search terms and results for all databases are presented in Supplementary material A.

#### Other literature

As AI is an area of intense commercial interest, data were also sought from sources beyond the academic literature to minimise publication bias (Supplementary material A). These resources have variable search function capabilities so, to enable a consistent approach, these repositories were searched using the subject headings from the search strategy of this review and all combinations thereof (‘artificial intelligence; ultrasound; anaesthesia’).

Two authors (JSB and MM) jointly searched the International Committee of Medical Journal Editors (ICMJE)-approved clinical trial registries for eligible studies on May 12, 2023. As the protocols and data from EudraCT are available via the EU Clinical Trials Register, the latter was searched in its place. In addition, the World Health Organization (WHO) clinical trials registry platform was searched on the same date.

The Ethos online library of doctoral theses was also jointly searched by two authors (JSB and MM, May 12, 2023), as were regulatory authority registries and competent authority websites of North America (US Food & Drug Administration; MAUDE, 510k, De Novo, and Medical Device Recall databases) and the UK (Medicines and Healthcare products Regulatory Agency).

To review centralised information in the clinical domain, two investigators (JSB, July 1, 2022, and JK, July 11, 2023) reviewed websites of prominent, international learned societies in regional anaesthesia for material relating to AI in UGRA: African Society for Regional Anesthesia; American Society of Regional Anesthesia & Pain Medicine; Asian and Oceanic Society of Regional Anaesthesia and Pain Medicine; European Society of Regional Anaesthesia and Pain Therapy; Latin American Society of Regional Anesthesia; Regional Anaesthesia UK.

Material from seven commercial organisations with products in the field were retrieved through independent search of the company websites by two investigators (JSB and MM, May 24–August 13, 2023). The included companies were GE Healthcare (Chicago, IL, USA), HiCura (Singapore), Intelligent Ultrasound (Cardiff, UK), Mindray (Shenzhen, China), Rivanna Medical (Charlottesville, VA, USA), Samsung (Suwon, South Korea), and SmartAlpha (Ankara, Tukey).

### Data extraction

#### Screening initial results

After removing duplicates, two investigators (JSB and MM) independently screened returned titles for inclusion in the study to determine whether they relate to AI, ultrasound, and regional anaesthesia. If the title was ambiguous, the source was included at this stage. In the event of disagreement between the two assessments, a third investigator (JK) reviewed to adjudicate.

#### Abstract review

One investigator (JSB) then reviewed the abstract/summary of all included sources for consideration of full-text review. Sources were retained if the abstract/summary related to AI, ultrasound, and regional anaesthesia (on human subjects).

#### Extraction of results

The full texts of all appropriate sources were reviewed (JSB) to ensure that each was still deemed suitable, then scrutinised to extract data on accuracy, utility, or both of the AI systems evaluated. The extracted data were electronically tabulated using a standardised data extraction form available in Supplementary material A. To ensure full scrutiny of each source, a software engineer (TH) initially reviewed all computer science/engineering papers. The lead investigator (JSB, clinician) then reviewed the extracted data from these sources along with the original publication, and assessed the clinically orientated sources, to aggregate all the information.

As formal assessment of study methodological quality is not recommended for scoping reviews,^14^ the investigators did not predefine what data would pertain to sono-anatomical structure identification accuracy or utility, or set any minimum outcome/reporting criteria. To maximise data capture, an inclusive approach was adopted when reviewing study items. As sources of commercial data may not include methodological reporting, this was not a prerequisite for inclusion.

Data were extracted from the included sources, and then the reference list for each was manually scrutinised for relevant cited literature (JSB). In addition, Google Scholar (Menlo Park, CA, USA) was used to identify pertinent citing literature (JSB). Further sources identified in this way were included for analysis.

## Results

### Selection of data sources

**Fig 1:**
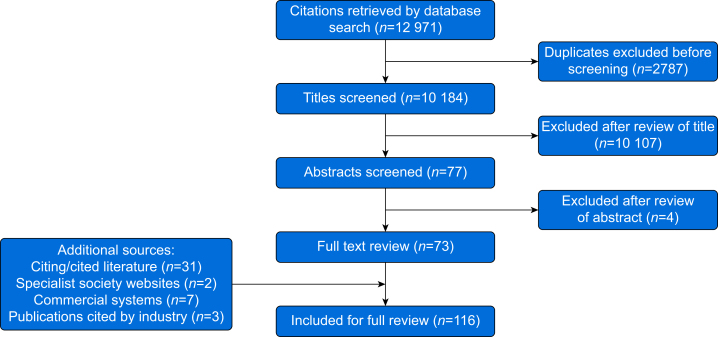
PRISMA flowchart showing the number of sources considered at each stage of the search and screening process. PRISMA, Preferred Reporting Items for Systematic Reviews and Meta-Analyses.

Figure 1 shows a flowchart of the search and screening process which details the number of sources considered at each stage. Overall, 116 sources were included for analysis; 84 pertaining to PNB, 30 to CNB, and two that addressed both areas. Where a source reported or summarised findings published elsewhere (e.g. review article or book chapter), data from the original source were used preferentially (*n*=93; 84 academic publications and nine commercial sources). Therefore, not all 116 sources have been cited in the manuscript, although additional sources are cited in Supplementary material B.

Iterative analysis of included sources identified six recurring themes in the data: AI methodology in use; output of AI systems; methods of evaluating accuracy; methods of evaluating utility; standardisation of study methodology/reporting; information on commercially available systems.

### Artificial intelligence methodology

#### Artificial intelligence and deep learning from 2016 to 2017

There is a notable change in the AI techniques used around 2016–17. Early approaches, most published before 2016–17, typically involved steps of feature extraction from the ultrasound image, followed by classification of these features, for both PNB^17^, ^18^, ^19^, ^20^, ^21^, ^22^, ^23^, ^24^, ^25^, ^26^, ^27^, ^28^ and CNB.^29^, ^30^, ^31^, ^32^, ^33^, ^34^, ^35^, ^36^, ^37^ Around this point, there has been an increase in published data where the dominant technique was deep learning for both PNB^9^^,^^11^^,^^12^^,^^38^, ^39^, ^40^, ^41^, ^42^, ^43^, ^44^, ^45^, ^46^, ^47^, ^48^, ^49^, ^50^, ^51^, ^52^, ^53^, ^54^, ^55^, ^56^, ^57^, ^58^, ^59^, ^60^, ^61^, ^62^, ^63^, ^64^, ^65^, ^66^, ^67^, ^68^, ^69^, ^70^, ^71^, ^72^, ^73^ and CNB,^7^^,^^74^, ^75^, ^76^, ^77^ though there are some exceptions.^78^, ^79^, ^80^ A number of sources do not explicitly state the techniques used; these are typically clinical case reports or (external) validation studies describing accuracy or efficacy^81^, ^82^, ^83^, ^84^, ^85^, ^86^, ^87^, ^88^, ^89^, ^90^, ^91^, ^92^, ^93^ and used alternative terms such as ‘automated’ or ‘intelligent’. However, these sources did identify the software in question (e.g. SpineNav3D software in the Accuro device, produced by Rivanna Medical). A full description of the AI, machine learning, and deep learning methodologies is provided in Supplementary material B.

#### Ground truth for model training/development

Ground truth refers to information known to be real and is used as the reference standard to train and test an AI technology. When training models in a supervised machine learning approach, raw data are presented alongside the ground truth and the model learns data patterns underlying this interpretation of the data. When evaluating models, the ground truth is used as a benchmark against which the models are assessed. It is therefore important to use a ground truth of the highest calibre to train models to the highest standard and assess them accurately.

In the available sources, a variety of human image interpretation was used as the ground truth. This included a range in the number of humans used in the ground truth from one,^7^^,^^23^, ^24^, ^25^^,^^31^^,^^32^^,^^34^, ^35^, ^36^^,^^38^^,^^46^^,^^49^^,^^58^^,^^60^^,^^67^^,^^72^^,^^77^ two,^8^^,^^9^^,^^19^^,^^21^^,^^26^^,^^30^^,^^33^^,^^44^^,^^61^ three,^51^, ^52^, ^53^^,^^63^^,^^65^ and up to 15.^48^ Some sources cited ‘multiple’ humans, used a plural term (e.g. experts), or simply did not state the number.^17^^,^^18^^,^^20^^,^^25^^,^^27^^,^^29^^,^^54^^,^^62^^,^^64^^,^^66^^,^^69^, ^70^, ^71^ Where multiple humans contributed to the ground truth, it was often unclear whether a single human assessed each image (with different humans assessing a different sample of the image dataset) or whether multiple humans assessed each image.

In addition to the varying number of humans contributing to the ground truth in model development, there was variation in the specialty and experience of those contributing. Sources described their ground truth human(s) as a volunteer,^64^ doctor/physician,^48^^,^^52^^,^^63^^,^^65^ sonographer,^7^^,^^30^, ^31^, ^32^, ^33^, ^34^, ^35^^,^^60^^,^^75^^,^^77^ clinical professional,^27^^,^^38^ or (regional) anaesthetist.^8^^,^^17^, ^18^, ^19^, ^20^, ^21^^,^^23^^,^^25^^,^^26^^,^^36^^,^^44^, ^45^, ^46^^,^^49^^,^^51^^,^^53^^,^^54^^,^^58^^,^^61^^,^^62^^,^^67^^,^^69^ The level of expertise of those providing the ground truth was described as trained,^64^ experienced,^21^^,^^30^, ^31^, ^32^, ^33^, ^34^^,^^46^^,^^48^^,^^52^^,^^65^ expert,^7^^,^^8^^,^^17^, ^18^, ^19^, ^20^^,^^25^, ^26^, ^27^^,^^33^^,^^35^^,^^45^^,^^53^^,^^58^^,^^61^^,^^66^^,^^67^^,^^69^^,^^71^^,^^77^ professional,^38^^,^^49^^,^^51^^,^^62^ specialist,^63^ or the level was unstated.^23^^,^^36^^,^^54^ However, sources did not typically define what was required to meet the definition of these levels and there is no universally recognised definition of ‘expert’ in regional anaesthesia.

For some sources, it is not clear how the ground truth for training data was obtained.^9^^,^^11^^,^^12^^,^^28^^,^^29^^,^^39^, ^40^, ^41^, ^42^, ^43^^,^^45^^,^^47^^,^^50^^,^^55^^,^^57^^,^^59^^,^^60^^,^^68^^,^^70^^,^^72^, ^73^, ^74^, ^75^, ^76^^,^^78^^,^^81^, ^82^, ^83^, ^84^, ^85^, ^86^, ^87^, ^88^, ^89^, ^90^, ^91^^,^^93^

### Model output

#### Peripheral nerve blockade

The most common output for models processing PNB ultrasound images was segmentation (the outline/colour overlay of features in the image).^9^^,^^11^^,^^12^^,^^19^^,^^20^^,^^22^^,^^23^^,^^25^^,^^27^^,^^36^^,^^38^, ^39^, ^40^, ^41^, ^42^, ^43^^,^^45^, ^46^, ^47^, ^48^, ^49^, ^50^, ^51^, ^52^, ^53^, ^54^, ^55^^,^^57^, ^58^, ^59^, ^60^^,^^63^, ^64^, ^65^, ^66^, ^67^, ^68^, ^69^^,^^71^^,^^72^^,^^78^^,^^79^ Less common outputs included the use of bounding boxes to identify the location of the structure,^8^^,^^17^^,^^18^^,^^26^^,^^61^ attempting to locate the centre of a nerve and placing expanding circular rings from the centre,^21^ or placing name tags over structures in question.^9^^,^^43^^,^^45^^,^^47^^,^^50^

Alternative methods proposed to support ultrasound scanning included ‘enhancing’ the image acquired,^56^ applying a ‘scan success’ indicator to the image,^9^^,^^43^^,^^45^^,^^47^^,^^50^ or classifying ultrasound images into the region scanned^70^^,^^73^ or optimal *vs* non-optimal views.^44^ Additional functions included creating a three-dimensional (3D) model of the structure in question,^24^^,^^28^ path planning for needle insertion,^20^ and guidance on probe movement.^22^

#### Central neuraxial blockade

The most common output for models processing CNB ultrasound images was classification/identification of vertebral level, the intervertebral space, or both.^30^, ^31^, ^32^, ^33^, ^34^^,^^75^, ^76^, ^77^^,^^81^, ^82^, ^83^, ^84^, ^85^, ^86^, ^87^, ^88^, ^89^, ^90^, ^91^^,^^93^ Distance from the skin to the posterior complex/epidural space was the second most common output of models applied to CNB ultrasound.^7^^,^^80^^,^^82^, ^83^, ^84^, ^85^, ^86^, ^87^, ^88^^,^^90^, ^91^, ^92^, ^93^ Other functions included identifying the optimal needle insertion point,^29^^,^^35^^,^^81^^,^^88^^,^^89^^,^^91^ structure identification (via segmentation or bounding boxes),^7^^,^^35^^,^^37^ or 3D reconstruction of the spine.^74^

### Accuracy evaluations

#### Peripheral nerve blockade

One approach to assessing accuracy of PNB model outputs involved assessing pixel-wise agreement between the ground truth and model output data, which was typically used for models that segment structures in the ultrasound image. The most common reporting metrics used to assess the overlap of enclosed areas (e.g. the outline of a blood vessel) were intersection over union (IoU; Jaccard similarity coefficient) and the Dice similarity coefficient.^8^^,^^17^, ^18^, ^19^, ^20^^,^^23^^,^^25^^,^^27^^,^^28^^,^^38^^,^^42^^,^^46^^,^^49^^,^^51^, ^52^, ^53^, ^54^, ^55^^,^^57^, ^58^, ^59^, ^60^^,^^62^, ^63^, ^64^, ^65^, ^66^^,^^68^^,^^69^^,^^71^^,^^79^ The Hausdorff metric/distance was used to compare the proximity of lines (e.g. a fascial plane).^19^^,^^20^^,^^25^^,^^27^^,^^28^^,^^42^^,^^53^^,^^69^ Whilst a pixel-wise approach clearly gives a detailed assessment of the proximity/coverage of one segmentation to another, there is no established threshold for this metric at which an AI system gives an ‘acceptable’ output. Furthermore, it gives little qualitative information on clinical relevance which is important as it may be more critical to accurately identify the exact border of a nerve or artery than a muscle.

An alternative approach takes an overall view of each whole structure by classifying the frequency of correct and incorrect prediction using true positive/negative and false positive/negative, accuracy, F-score, precision, recall, sensitivity, specificity, and area under the curve.^18^^,^^19^^,^^39^^,^^44^^,^^54^^,^^55^^,^^58^^,^^59^^,^^61^^,^^67^^,^^69^^,^^72^ Bowness and colleagues^39^ used the majority opinion from a panel of three experts to determine whether an AI structure identification was a true positive/negative etc; whilst this method would appear to incorporate clinical context, it is a pooled subjective opinion. The remaining publications used a frequency classification for each whole structure and based the decision of correct identification on a cut-off in the pixel-wise assessment. The definitions of correct prediction included IoU of >0.5,^8^^,^^17^^,^^18^^,^^46^^,^^65^ ≥25% pixel overlap,^58^^,^^67^ and subjective rating for a range of IoU values (e.g. 0.41–0.6=fairly good precision; 0.61–0.8=good precision),^38^ whilst others did not define the threshold of effective segmentation.^54^^,^^55^

Other methods of assessing/reporting accuracy were used infrequently, including subjective human assessment without providing data (‘performed as claimed’^50^ or ‘the identification of regional anatomy and scanning were successful’^47^) and a subjective 1–5 Likert scale rating of accuracy.^9^

It is important to note that ultrasound scans used for the assessments were collected on different subjects, using different machines, and in different body regions/structures. A limited number of accuracy assessments used multiple structure classes (e.g. nerve, artery, bone) over multiple body regions,^9^^,^^39^^,^^42^ whilst some covered multiple structure classes in a single region (e.g. supraclavicular level brachial plexus or thoracic paravertebral block).^22^^,^^23^^,^^27^^,^^44^^,^^46^^,^^49^^,^^53^^,^^58^^,^^62^^,^^67^^,^^78^ Other sources used a single structure class (e.g. nerve) from multiple regions,^8^^,^^36^^,^^54^ or a single structure in a single region, such as the median nerve in the forearm,^17^, ^18^, ^19^, ^20^^,^^61^^,^^69^ the femoral artery,^28^^,^^72^ the femoral nerve,^38^^,^^65^ or the sciatic nerve.^21^^,^^25^^,^^48^^,^^57^ Finally, some studies did not fully define the region/structures being assessed; often simply naming the ‘brachial plexus’,^51^^,^^52^^,^^55^^,^^59^^,^^60^^,^^63^^,^^64^^,^^66^^,^^68^^,^^71^^,^^79^ or not stating the region(s) or structure(s) at all.^47^^,^^50^

Ultimately, studies failed to consistently use the same metrics to compare segmentation of the same structures on the same ultrasound scans. This makes it very difficult for clinicians to effectively compare performance of one model/approach to others.

#### Central neuraxial blockade

Assessment of accuracy for CNB models was more aligned than the approaches for assessing PNB model accuracy, which involved a greater element of human judgement/interpretation. The most common method was correlating the distance from skin to posterior complex/epidural space as predicted by the model to that observed on manual interpretation of ultrasound,^32^^,^^34^^,^^84^^,^^88^^,^^93^ depth of loss of resistance during needle insertion,^80^^,^^82^^,^^85^^,^^86^ or both.^37^^,^^83^^,^^92^ All reported good correlation between human depth assessment and AI depth assessment on the ultrasound image, and loss of resistance during needle insertion to be deeper than that calculated on ultrasound (by human or AI).

Accuracy in determining the level of the intervertebral space in view was assessed by five studies. All report ≥84% agreement in AI and human identification of vertebral level based on ultrasound interpretation,^30^^,^^34^^,^^75^, ^76^, ^77^ whereas a single study reported 70% agreement between palpation and AI-ultrasound.^75^ Similarly, four studies reported ≥93% accuracy in AI identification of lumbar ultrasound images showing an intervertebral space *vs* a spinous process, though this approach did not determine which level was in view.^29^^,^^31^, ^32^, ^33^

The ultrasound body regions used for CNB approaches were more consistent than for PNB. Nineteen sources used ultrasound scans of the lumbar spine^7^^,^^29^, ^30^, ^31^, ^32^, ^33^, ^34^, ^35^^,^^37^^,^^75^, ^76^, ^77^^,^^80^^,^^83^^,^^84^^,^^86^^,^^88^^,^^90^^,^^92^^,^^93^ and two utilised the thoracolumbar spine,^74^^,^^82^ whereas one did not specifically state which region was seen (though use of the lumbar region can be deduced as it is a case report of a patient undergoing total knee arthroplasty).^85^

#### Ground truth for accuracy assessments

Many studies reported results of testing or internal validation in which a proportion of the initial dataset was partitioned from the training data and used to assess models at the end of training. External validation is the subsequent assessment of these models, using data collected at a different time point, location, population, or all. It is used to further evaluate model performance and assess generalisability to the wider population (or different populations). Only one source explicitly stated it was an external validation study.^39^

Of the sources reporting accuracy data for PNB models, seven described external validation studies. The standard (ground truth) used for these studies was described as a single ‘expert anaesthetist’,^22^ two ‘regional anaesthesia experts’,^20^ ‘an experienced anaesthetist and a radiologist’,^9^ or ‘three UGRA experts’.^39^ One study compared AI model performance to that of 19 ‘experts’.^42^ It is not stated in/clear from two sources who was providing the gold standard (ground truth) for these assessments.^47^^,^^50^

Twelve sources for CNB models described external validation studies of accuracy. The gold standard (ground truth) for these studies were an anaesthetist,^29^^,^^30^^,^^80^^,^^82^, ^83^, ^84^^,^^86^^,^^92^ clinician,^88^^,^^93^ ‘provider experienced with freehand ultrasound’,^75^ or is not stated.^85^ Their level of expertise was described as experienced,^30^^,^^88^^,^^93^ novice and senior/expert,^82^^,^^84^ resident/fellow/attending,^83^^,^^86^^,^^92^ or unstated.^29^^,^^75^^,^^80^^,^^85^ The number of humans contributing to this gold standard (ground truth) was one,^30^^,^^75^^,^^83^^,^^86^^,^^88^^,^^92^^,^^93^ two,^84^ or unstated.^29^^,^^85^

### Utility evaluations

#### Peripheral nerve blockade

Utility evaluations for PNB models can be broadly divided into assessments based on subjective opinion (*n* =10) or on clinical outcomes (*n*=4).

In a non-clinical or simulation environment, Bowness and colleagues^11^ have published a number of studies in which a panel of three UGRA experts rated the AI colour overlay on recorded ultrasound videos (7.87–8.69/10); assessed that it would be helpful in determining the correct view and identifying anatomical structures of interest in >99% of cases^11^; determined the accuracy rate of structure identification to be 93.5%^39^; and reduced the risk of unwanted needle-induced trauma or block failure in 62.9–86.4% of cases.^39^ In other studies this group has sought feedback from 30 users to determine use of this system for UGRA experts (teaching) and non-experts (learning/clinical practice),^12^ and assessed 21 UGRA non-experts to demonstrate superior scanning performance (on volunteers) with the use of an AI colour overlay on real-time ultrasound.^40^ Cai and colleagues^48^ demonstrated higher self-rating and expert assessment scores amongst 40 anaesthetic trainees when performing PNBs after teaching with an AI-based nerve identification system, whereas Erdem and colleagues^43^ describe that 40 anaesthetists reported reduced chance of complications, increased chance of block success, and that the system was useful as a training tool in UGRA. Other studies report that anaesthetists described the assistive AI technology to be helpful in the identification of key anatomical structures and in training,^45^ and that all non-experts in UGRA considered assistive AI to be a desirable and useful tool.^24^ Other published subjective opinion includes a 20–30% reduction in time required for UGRA procedures and a reduction in needle pass attempts (with no underlying data provided in this source),^50^ and a report which proposed that assistive AI may improve block success, reduce the number of needle attempts, improve the satisfaction of patient/practitioner, and reduce the volume of local anaesthetic required (again without providing underlying data).^47^

Clinical assessments include a secondary analysis of data collected during a service evaluation by Bowness and colleagues,^41^ who reported an increase in the delivery of UGRA to trauma patients when assistive AI for ultrasound scanning was available. In 28 days before the introduction of the AI system, 71 PNBs were performed amongst 207 eligible cases, compared with 93 for 193 eligible cases after (*P*=0.036)—with no observed decrease in efficacy or increase in complication rate.^41^ Other clinical outcome data for PNB systems report that UGRA performed with AI-enhanced ultrasound imaging achieved a quicker performance of UGRA, superior motor block, and better analgesia in scapula fracture surgery.^56^ Cai and colleagues^48^ report reduced paraesthesia during the first month of performing sciatic nerve blocks after teaching with an AI-based nerve identification system, though there was no difference in the rate of pain during injection, perforation of blood vessels or block success. Wang and colleagues^78^ report reduced postoperative analgesic requirement and cognitive dysfunction/delirium after AI-assisted lumbosacral plexus block compared with systemic analgesia for hip arthroplasty surgery.

The above sources again report evaluations for PNB for varying body regions. Some studies covered PNB for three or more body regions,^11^^,^^12^^,^^39^, ^40^, ^41^^,^^43^ whereas one source covered the supraclavicular and adductor canal blocks,^45^ three covered a single PNB region (lumbar plexus,^78^ ‘scapula region nerve block’,^56^ and the sciatic nerve^48^), and two did not state the PNB region assessed.^47^^,^^50^

#### Central neuraxial blockade

Utility evaluations for CNB models can be broadly divided into assessments based on technical success of the procedure (*n*=13) or on clinical outcomes (*n*=4).

Technical success criteria included reporting needle insertion attempts or first pass success; three sources reported fewer attempts/higher success rates,^80^^,^^90^^,^^91^ two reported no statistically significant difference,^87^^,^^89^ and three sources simply reported rate of first pass success using AI assistance (87.23%, 79.1%, and 92.0%, respectively) without comparison to no AI assistance.^86^^,^^88^^,^^93^ Two sources were case reports of successful epidural catheter insertion in patients living with obesity.^81^^,^^85^ A second criterion for technical success was the number of needle directs, with three sources reporting fewer redirects were required with AI assistance.^82^^,^^90^^,^^91^ As with utility evaluations of PNB models, time taken for the procedure/to identify the appropriate view (intervertebral space) was assessed in a number of sources. This showed variable results, with some reporting increased time,^87^^,^^91^ no difference,^82^^,^^89^ a mixed picture,^75^^,^^80^ or reduced time required.^90^ Two sources report the time taken for the procedure, but did not provide a comparison with an alternative technique (28 s and 31 s, respectively).^30^^,^^85^

In clinical outcome studies, no significant difference in patient satisfaction was associated with use of AI-assisted ultrasound scanning for CNB,^87^ or failed block and adverse events,^91^ though a lower incidence of paraesthesia and lower back pain have been reported elsewhere.^80^^,^^90^

As might be expected, the body region used to evaluate AI assistance for ultrasound scanning in CNB was more consistent than for PNB. Eleven sources report using ultrasound scans of the lumbar spine^30^^,^^75^^,^^80^^,^^81^^,^^86^, ^87^, ^88^, ^89^, ^90^, ^91^^,^^93^ and one utilised the thoracolumbar spine,^82^ whilst one did not specifically state which region was seen though use of the lumbar region can be deduced as it is a case report of a patient undergoing total knee arthroplasty.^85^

### Standardisation of study methodology and reporting

An additional limitation to comparing evaluations of this technologies is that reporting varies considerably; only 32/93 (34.4%) reviewed sources reported demographic data for the subjects assessed in these studies.^7^^,^^9^^,^^12^^,^^21^^,^^22^^,^^29^^,^^30^^,^^35^^,^^37^, ^38^, ^39^, ^40^, ^41^, ^42^^,^^44^^,^^46^^,^^52^^,^^53^^,^^74^^,^^75^^,^^78^^,^^80^, ^81^, ^82^, ^83^, ^84^, ^85^^,^^88^^,^^90^, ^91^, ^92^, ^93^ This is important as demographic factors such as age (muscle atrophy) and obesity affect the appearance of anatomical structures on the ultrasound image.^94^ These descriptions were themselves inconsistent; such as reporting age and gender, but not BMI,^78^ or reporting only mean age/BMI without ranges.^74^ Importantly, older and obese patients were underrepresented with only 18/32 (56.3%) sources reporting demographic data which included subjects aged >60 yr, BMI ≥30 kg m^−2^, or both.^12^^,^^37^, ^38^, ^39^, ^40^, ^41^, ^42^^,^^52^^,^^80^^,^^81^^,^^83^, ^84^, ^85^, ^86^^,^^88^^,^^90^, ^91^, ^92^

Models for CNB ultrasound were most commonly assessed in the obstetric population, with 14 sources reporting data in this cohort.^31^, ^32^, ^33^, ^34^^,^^37^^,^^75^^,^^81^^,^^83^^,^^84^^,^^86^^,^^88^^,^^90^^,^^92^^,^^93^ Despite CNB being a common technique throughout anaesthesia, only four sources included non-obstetric patients,^80^^,^^82^^,^^85^^,^^91^ whilst four utilised healthy volunteers,^29^^,^^30^^,^^35^^,^^77^ and three sources did not identify the subject population being scanned.^7^^,^^74^^,^^76^

Only six sources reported their data according to recognised reporting standards,^40^^,^^42^^,^^82^^,^^86^^,^^88^^,^^92^ including the CONSORT-AI,^95^ CONSORT,^96^ DECIDE-AI,^97^ and STROBE^98^ guidelines. Of these, only CONSORT-AI and DECIDE-AI are AI-specific. As they were published in 2020 and 2022, respectively, time will tell whether future studies are reported in a more consistent manner and in line with these guidelines.

Finally, it is good practice in the field of AI to make training and testing/validation data publicly available.^99^, ^100^, ^101^ However, only 15 sources used publicly available data or shared their data when publishing.^48^^,^^49^^,^^51^^,^^55^^,^^59^, ^60^, ^61^^,^^63^, ^64^, ^65^, ^66^^,^^68^^,^^71^^,^^74^^,^^79^

### Commercially available systems

Seven AI systems are commercially available to support ultrasound scanning in UGRA, five for PNB and two for CNB (Table 1).

**Table 1:** Commercial systems and accompanying information. AI, artificial intelligence; CNB, central neuraxial blockade; PNB, peripheral nerve blockade. ∗Company approached for information on regulatory approval in UK and EU but not provided.

System name Manufacturer	Approved as a medical device	Description	Company claims	Sources of data on performance
cNerve GE Healthcare (Chicago, IL, USA)	UK/EU (Unknown∗) USA (June 2022)	Colour overlay segmentation of interscalene to supraclavicular-level brachial plexus, femoral, and popliteal-level sciatic nerve	*‘Helps detect and track nerves in 99% of cases’*	Commercial^102^
Nerveblox SmartAlpha (Ankara, Turkey)	UK/EU (May 2021)	Colour overlay of blood vessels, bone, muscles, nerves, and fascia/serosa for 12 PNBs	No specific claims regarding accuracy *‘ … gives anesthesiologists extra confidence … ’* *‘ … help anesthesiologists practice PNB faster.’*	Commercial^105^ Academic^9^^,^^43^^,^^47^^,^^50^
NerveTrack Samsung (Suwon, South Korea)	UK/EU (February 2021) USA (May 2021)	Bounding box around median and ulnar nerves in the forearm	*‘ … can detect the median and ulnar nerve with reasonable accuracy … ’* *‘ … efficiency of nerve detection … ’* is claimed to be 4.4/5 with *vs* 3.8/5 without (*P*<0.0001) *‘ … reduce scanning time significantly, from 24.7 s to 8.2 s.’*	Commercial^10^^,^^104^
ScanNav Anatomy Peripheral Nerve Block Intelligent Ultrasound (Cardiff, UK)	UK/EU (April 2021) USA (October 2022)	Colour overlay of blood vessels, bone, muscles, nerves, and fascia/serosa for 10 PNBs	*‘ … enhance the accuracy and standardization of ultrasound image interpretation … ’* *‘ … help tip the balance of safety and confidence in favour of performing regional anaesthesia.’*	Commercial^106^ Academic^6^^,^^11^^,^^12^^,^^39^, ^40^, ^41^
Smart Nerve Mindray (Shenzhen, China)	UK/EU (Unknown∗) USA (November 2021)	Colour overlay segmentation of interscalene to supraclavicular-level brachial plexus nerves	No specific claims regarding accuracy *‘ … automatically recognise the brachial plexus and highlight the nerve, increasing clinical confidence and reducing procedure time … ’*	Commercial^103^
Accuro Rivanna Medical (Charlottesville, VA, USA)	UK/EU (June 2016) USA (March 2014)	Provides guidance for CNB (spinal and epidural) in the thoracic and lumbar regions of the spine using *‘AI-enabled SpineNav3D image recognition’*. Software identifies intervertebral space on ultrasound image and hardware identifies point of needle insertion on the skin.	*‘Improve the safety, speed, and efficiency of epidural and spinal anesthesia.’* *‘ … accurately identifies the epidural location with success rates exceeding 94%, Accuro gives you confidence … ’* *‘Clinically proven to:* - *Increase first attempt success* - *Reduce needle passes* - *Reduce placement times* - *Significantly increase patient satisfaction and pain control’* *‘Collectively, these benefits significantly reduce the cost of care’*	Commercial^107^^,^^108^ Academic^81^, ^82^, ^83^, ^84^, ^85^, ^86^, ^87^^,^^89^, ^90^, ^91^, ^92^
uSine HiCura Medical (Singapore)	UK/EU (August 2023) Singapore (July 2022)	Additional screen labels spinous processes and intervertebral spaces on lumbar ultrasound scan, and measures epidural depth *‘ … machine learning algorithm allows automatic identification of spinal landmarks during ultrasound scan.’*	*‘ … alerts anaesthetist in real-time when the right location and right angle are reached.’* *‘ … achieved very high [92%] first-attempt puncture success rate.’* *‘ … safe and effective and promotes a reduction of procedural time, better clinical outcomes and improves patient satisfaction.’*	Commercial^109^ Academic^88^^,^^93^

#### Peripheral nerve blockade

cNerve (GE Healthcare)^102^ and Smart Nerve (Mindray)^103^ are systems incorporated into the manufacturers' ultrasound machines, which segment peripheral nerves to produce a (yellow) colour overlay over them. NerveTrack (Samsung Medison, Suwon, South Korea)^10^^,^^104^ is also integrated into the manufacturer's machine, but produces bounding boxes around the median and ulnar nerves of the forearm. Nerveblox (SmartAlpha)^105^ and ScanNav Anatomy Peripheral Nerve Block (Intelligent Ultrasound)^106^ are external devices, which can be connected to an ultrasound machine to provide an additional display with an AI-generated colour overlay of key structures for a number of PNBs. Numerous studies have been published on the latter device,^12^^,^^39^, ^40^, ^41^, ^42^ all of which are included in this review.

#### Central neuraxial blockade

Accuro (Rivanna Medical) is a handheld device with an integrated ultrasound machine,^107^^,^^108^ whereas uSine (HiCura) is an external device which is connected to the ultrasound machine.^109^ Both systems aim to support identification of the vertebral level, intervertebral space, and depth to target. The former system incorporates hardware to mark the patient's skin with the optimal needle insertion site.

#### Conflicts of interests

As in any field, declaring sources of funding and conflicts of interest is essential to transparent reporting of research. Work in AI has a potential for commercial value, thus it is particularly pertinent in this setting. Of the 23 academic studies which directly relate to a commercial product (see Table 1), 10 declare funding for research (or in-kind support), industry affiliations, or both in the conflict of interests statement.^6^^,^^11^^,^^12^^,^^39^, ^40^, ^41^^,^^86^^,^^90^, ^91^, ^92^ Two report these affiliations in the limitations section of the discussion.^39^^,^^40^

## Discussion

AI in healthcare is an area of intense and growing interest;^110^ the worldwide AI healthcare market projected to reach $200 billion by 2030^111^ and the number of regulatory approvals for AI-based medical devices is steadily increasing.^112^ Anaesthesia is a data-rich specialty, with a heavy reliance on technology, but there are relatively few AI devices approved for use in this field of medicine compared with others such as radiology and cardiology.^113^

This scoping review has surveyed literature across multiple disciplines and identified key findings. Firstly, the dominant technique is deep learning, which is used in virtually all publications since 2017. In addition, model outputs show consistency; most commonly sono-anatomical structure segmentation for PNB or identification of intervertebral space and depth to target for CNB. Secondly, and conversely, methods for evaluating accuracy and utility originate in multiple disciplines and show gross heterogeneity with a notable lack of clinical data. Thirdly, reporting is inconsistent amongst academic publications and data on device performance information from commercial organisations is scarce—often produced by clinicians in subsequent studies rather than made available by the companies from their regulatory approval studies.

It is possible that heterogenous and unstructured research literature remains a barrier to AI being successfully incorporated into clinical anaesthetic practice. The uniformity in AI techniques now used, and consistency in model outputs, should facilitate further standardisation. This will engender greater understanding of this technology and support appropriate adoption in clinical practice. Despite the rapid growth in the field, this should provide cause for optimism that appropriate structure can be implemented successfully.

However, this is an interdisciplinary field, and the discordant approaches of clinical medicine, technology, and industry are striking. This is evident in the anatomical structures that groups/companies chose to identify, how models are trained (e.g. source of ground truth), how accuracy is validated (e.g. metric used), and how utility is assessed. In addition, there is still a paucity of data on patient outcomes and institutional impact. Standardisation has become a common feature of UGRA.^114^, ^115^, ^116^, ^117^ Whilst initial efforts have been made to address this in AI,^118^^,^^119^ it has not yet been realised and the authors believe that this limitation will continue to impede clinical implementation of AI in UGRA until it is addressed.

Enthusiasm to share novel findings in this exciting and fast-moving field is understandable, but differences in reporting also hinder reliable comparison of models/systems and limit a true understanding of the state of the art. Furthermore, as companies are not incentivised to share data from their regulatory filings, there is variability in approaches to regulatory approval and a lack of understanding of the relative performance of approved devices.

**Fig 2:**
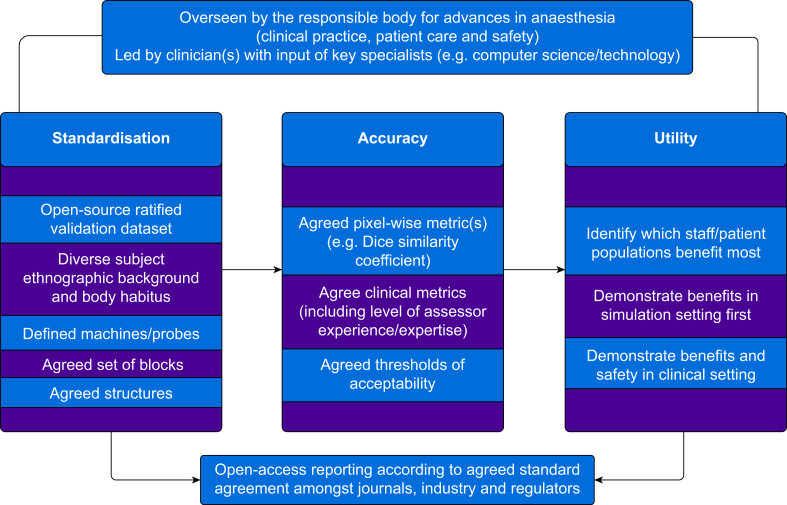
Conceptual overview of a structured framework for the validation of accuracy and clinical utility of AI technology which assists ultrasound scanning in UGRA. AI, artificial intelligence; UGRA, ultrasound-guided regional anaesthesia.

We propose that a structured framework be developed for validating accuracy and clinical utility of AI which assists ultrasound scanning in UGRA (Fig. 2). This should include which sono-anatomical structures systems identify for specific procedures, a standardised method for both pixel-wise and clinical assessment of accuracy, clearly identified measures of clinical utility, demonstration of clinical impact, and standardised reporting.^120^ Clinical bodies should lead the development of this framework, to ensure clinically relevant pain points are addressed, and ensure contribution from technology experts, clinical medicine, industry and regulatory bodies, and patient and public involvement. Developing an open-access dataset of representative ultrasound scans for all parties to utilise in validation studies will be a key element to facilitating standardised evaluation. Industry should be encouraged to utilise this and publish their data. Standardised reporting of these evaluations will further aid dissemination of knowledge, aided by a non-critical view of declared potential conflicts of interest.

Ultrasound revolutionised UGRA in an unstructured manner has led to great progress, but barriers in the field remain as some techniques to assist with ultrasound image interpretation remain unvalidated (e.g. hydrolocation).^4^ Recent initiatives are attempting to retrospectively provide structure.^121^ AI will revolutionise medicine,^110^ and potentially UGRA; adopting this technology in a structured manner will provide the optimal opportunities to harness its full potential.

### Conclusions

This scoping review has identified gross heterogeneity and poor reporting across the literature pertaining to AI for the identification of anatomical structures on ultrasound in regional anaesthesia. This is an important barrier to developing the field and implementing AI technologies within clinical anaesthetic practice. The situation can only be improved by clinicians, scientists, and industry working together to standardise our approach to understanding these systems, to optimise use in ultrasound-guided regional anaesthesia.

## Authors’ contributions

Study concept and design: JSB, DM, KEB, HH

Design and conduct of literature search: JSB, NT, MM

Review of search results and search of grey literature: JSB, MM, JK

Data extraction: JSB, TH

Manuscript preparation and editing: JSB

Manuscript review & approval: all authors

## Declarations of interest

JSB is a senior clinical advisor for Intelligent Ultrasound, receiving research funding and honoraria. KE has received research, honoraria, and educational funding from Fisher and Paykel Healthcare, GE Healthcare, PAION, and Ambu. MM was formerly and TH is currently employed by Intelligent Ultrasound. JAN is a senior scientific advisor for Intelligent Ultrasound.

## Figures and Tables

**Figure S1:**
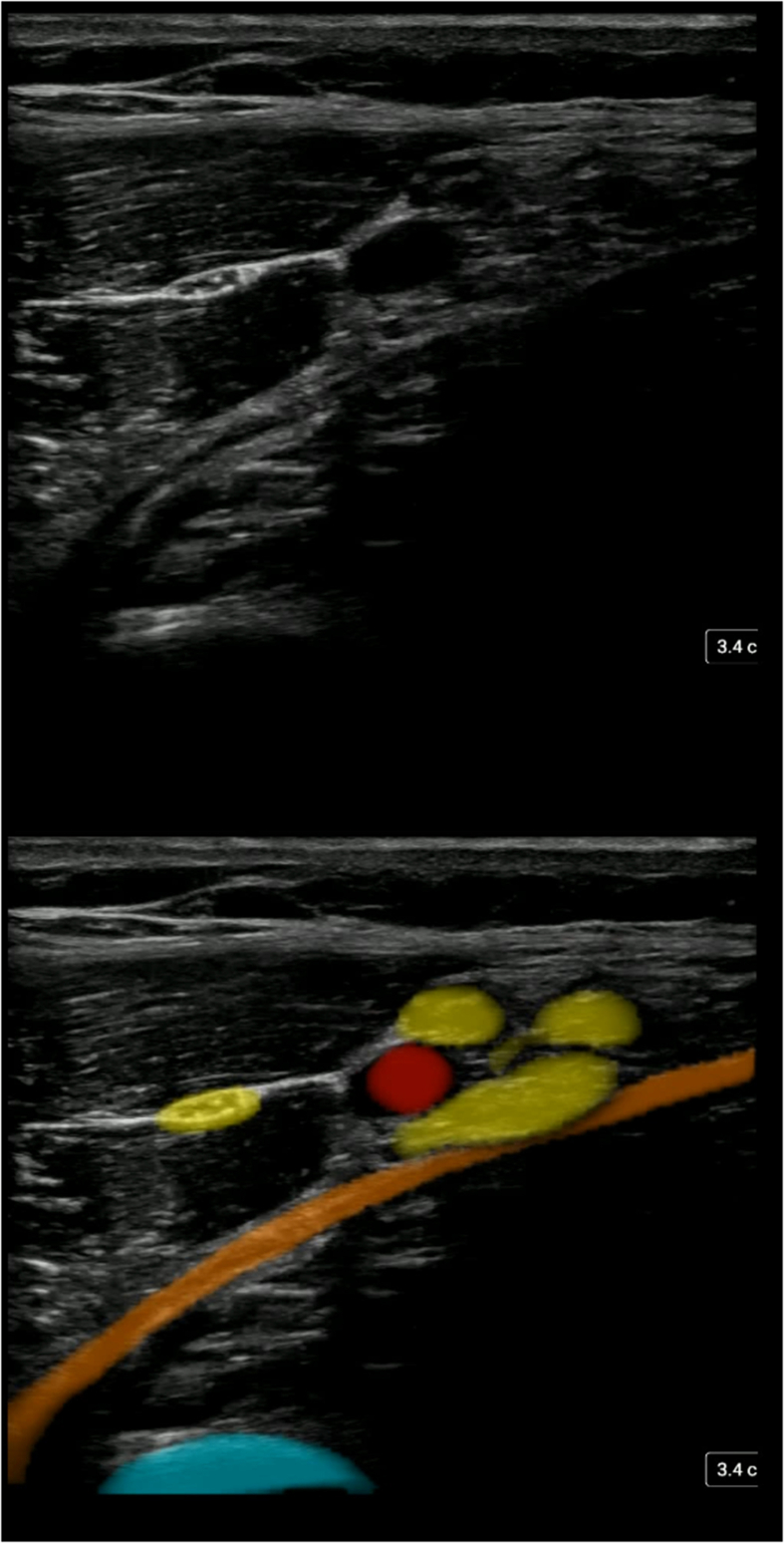


**Figure S2:**