# Supplementary material for: Artificial intelligence for ultrasound scanning in regional anaesthesia: a scoping review of the evidence from multiple disciplines
Source: Br J Anaesth. 2024 Mar 5;132(5):1049–62. doi: 10.1016/j.bja.2024.01.036 (PMC11103083; doi:10.1016/j.bja.2024.01.036)
Supplement: Multimedia component 1 [file mmc1.docx]

**Artificial Intelligence for Ultrasound Scanning in Regional Anaesthesia: A Scoping Review of the Evidence from Multiple Disciplines**

**SUPPLEMENTARY MATERIAL A – SEARCH STRATEGY/RESULTS & DATA EXTRACTION FORM**

**Authors**

James S Bowness^1,2^, David Metcalfe^3,4^, Kariem El-Boghdadly^5,6^, Neal Thurley^7^, Megan Morecroft^8^, Thomas Hartley^9^, Joanna Krawczyk^2^, J Alison Noble^10^, Helen Higham^1,11^

**Author Institutions**

1. Nuffield Department of Clinical Neurosciences, University of Oxford
2. Department of Anaesthesia, Aneurin Bevan University Health Board
3. Nuffield Department of Orthopaedics, Rheumatology & Musculoskeletal Sciences, University of Oxford
4. Emergency Medicine Research in Oxford (EMROx), Oxford University Hospitals NHS Foundation Trust
5. Department of Anaesthesia and Peri-operative Medicine, Guy’s & St Thomas’s NHS Foundation Trust
6. Centre for Human and Applied Physiological Sciences, King’s College London
7. Bodleian Health Care Libraries, University of Oxford, UK
8. Faculty of Medicine, Health & Life Sciences, University of Swansea
9. Intelligent Ultrasound
10. Institute of Biomedical Engineering, University of Oxford
11. Nuffield Department of Anaesthesia, Oxford University Hospitals NHS Foundation Trust

**Corresponding Author**

James S Bowness

OxSTaR Centre, Nuffield Division of Anaesthetics, Nuffield Department of Clinical Neurosciences, John Radcliffe Hospital, Oxford, OX3 9DU

Tel: 0044 1865 231 510. E-mail: [james.bowness@jesus.ox.ac.uk](mailto:james.bowness@jesus.ox.ac.uk)

**Table 1. Search Strategy and Terms**

| **Artificial Intelligence** | **AND** | **Ultrasound** | **AND** | **Regional Anaesthesia** |
| --- | --- | --- | --- | --- |
| “artificial intelligence”.ti,ab. |  | ultraso*.ti,ab. |  | anaesthe*.ti,ab. |
| OR |  | OR |  | OR |
| AI.ti,ab. |  | sonog*ti.ab |  | anesthe*.ti,ab. |
| OR |  |  |  | OR |
| “computer vision”.ti,ab. |  |  |  | regional.ti,ab. |
| OR |  |  |  | OR |
| “machine intelligence”.ti,ab. |  |  |  | nerve*.ti,ab. |
| OR |  |  |  | OR |
| “deep learning”.ti,ab. |  |  |  | block*.ti,ab. |
| OR |  |  |  | OR |
| “machine learning”.ti,ab. |  |  |  | spinal.ti,ab. |
| OR |  |  |  | OR |
| “neural network*”.ti,ab. |  |  |  | epidural.ti,ab. |
| OR |  |  |  | OR |
| “computer aid*”.ti,ab. |  |  |  | “central neuraxial”.*ti,ab. |
| OR |  |  |  |  |
| automat*.ti,ab. |  |  |  |  |
| OR |  |  |  |  |
| segment*.ti,ab. |  |  |  |  |

**Table 1. Search Results for Medline (OvidSP) [1946 – present]; Total Records 3,022**

| 1 | exp Artificial Intelligence/ | 170051 |
| --- | --- | --- |
| 2 | artificial intelligence.mp. | 55287 |
| 3 | ai.mp. | 45034 |
| [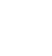](http://ezproxy-prd.bodleian.ox.ac.uk:2481/ovid-a/ovidweb.cgi?&S=GPKGFPOMHBEBKFBDIPAKNGEHIFAAAA00&R=3&Search+Annotations+Options=SA)   \| 4 \| \| --- \| | deep learning.mp. | 46847 |
| 5 | machine learning.mp. | 91022 |
| 6 | automat*.mp. | 313599 |
| 7 | segment*.mp. | 472509 |
| 8 | computer vision.mp. | 7611 |
| 9 | machine intelligence.mp. | 274 |
| 10 | **computer aid*.mp.** | 38944 |
| 11 | exp neural networks, computer/ | 57567 |
| 12 | neural network*.mp. | 103664 |
| 13 | Automation/ | 19993 |
| 14 | 1 or 2 or 3 or 4 or 5 or 6 or 7 or 8 or 9 or 10 or 11 or 12 or 13 | 1035688 |
| 15 | ultrasonography/ or ultrasonography, interventional/ | 225773 |
| 16 | ultraso*.mp. | 578492 |
| 17 | sonog*.mp. | 64766 |
| 18 | 15 or 16 or 17 | 593176 |
| 19 | anesthesia/ or anesthesia, conduction/ or anesthesia, epidural/ or anesthesia, local/ or anesthesia, spinal/ or nerve block/ or autonomic nerve block/ or brachial plexus block/ or cervical plexus block/ | 131547 |
| 20 | anaesthe*.mp. | 115423 |
| \| 21 \| \| --- \| | anesthe*.mp. | 436057 |
| 22 | regional.mp. | 374173 |
| 23 | block*.mp. | 940675 |
| 24 | spinal*.mp. | 443802 |
| 25 | epidural*.mp. | 56042 |
| 26 | central neuraxial*.mp. | 226 |
| 27 | 19 or 20 or 21 or 22 or 23 or 24 or 25 or 26 | 2108603 |
| 28 | 14 and 18 and 27 | 3022 |

**Table 2. Search Results for Embase (OvidSP) [1974 – present]; Total Records 7,902**

| 1 | exp *artificial intelligence/ | 38745 |
| --- | --- | --- |
| 2 | artificial intelligence.mp. | 64562 |
| 3 | ai.mp. | 61088 |
| 4 | deep learning.mp. | 58705 |
| 5 | machine learning.mp. | 127611 |
| 6 | automat*.mp. | 435276 |
| 7 | segment*.mp. | 686045 |
| 8 | computer vision.mp. | 8876 |
| 9 | machine intelligence.mp. | 315 |
| \| 10 \| \| --- \| | computer aid*.mp. | 49074 |
| 11 | exp *artificial neural network/ | 43850 |
| 12 | neural network*.mp. | 133232 |
| 13 | exp *automation/ | 44275 |
| 14 | 1 or 2 or 3 or 4 or 5 or 6 or 7 or 8 or 9 or 10 or 11 or 12 or 13 | 1405039 |
| 15 | exp *echography/ | 248939 |
| 16 | ultraso*.mp. | 789329 |
| 17 | sonog*.mp. | 95479 |
| 18 | 15 or 16 or 17 | 926724 |
| 19 | exp *anesthesia/ | 176039 |
| 20 | anaesthe*.mp. | 168596 |
| 21 | anesthe*.mp. | 621838 |
| 22 | regional.mp. | 448428 |
| 23 | block*.mp. | 1431609 |
| 24 | spinal*.mp. | 500827 |
| 25 | epidural*.mp. | 88584 |
| 26 | central neuraxial*.mp. | 556 |
| 27 | 19 or 20 or 21 or 22 or 23 or 24 or 25 or 26 | 2836879 |
| 28 | 14 and 18 and 27 | 7902 |

**Table 3. Search Results for CINAHL (EBSCO) [1981 – present]; Total Records 1,192**

| # | Query | Results |
| --- | --- | --- |
| S1 | (MH "Artificial Intelligence+") | 28,510 |
| S2 | TI "artificial intelligence" OR AB "artificial intelligence" | 7,662 |
| S3 | TI ai OR AB ai | 9,364 |
| S4 | TI "deep learning" OR AB "deep learning" | 4,995 |
| S5 | TI "machine learning" OR AB "machine learning" | 12,193 |
| S6 | TI automat* OR AB automat* | 47,428 |
| S7 | TI segment* OR AB segment* | 59,587 |
| S8 | TI "computer vision" OR AB "computer vision" | 501 |
| S9 | TI "machine intelligence" OR AB "machine intelligence" | 37 |
| S10 | TI "computer aid*" OR AB "computer aid*" | 4,437 |
| S11 | (MH "Neural Networks (Computer)") | 3,633 |
| S12 | TI "neural network*" OR AB "neural network*" | 7,179 |
| S13 | (MH "Automation+") | 16,365 |
| S14 | S1 OR S2 OR S3 OR S4 OR S5 OR S6 OR S7 OR S8 OR S9 OR S10 OR S11 OR S12 OR S13 | 152,068 |
| S15 | (MH "Ultrasonography+") | 115,348 |
| S16 | TI ultraso* OR AB ultraso* | 98,710 |
| S17 | TI sonog* OR AB sonog* | 14,617 |
| S18 | S15 OR S16 OR S17 | 175,067 |
| S19 | (MH "Anesthesia+") | 48,078 |
| S20 | TI anaesthe* OR AB anaesthe* | 27,895 |
| S21 | TI anesthe* OR AB anesthe* | 53,743 |
| S22 | TI regional OR AB regional | 67,216 |
| S23 | TI block* OR AB block* | 92,275 |
| S24 | TI spinal* OR AB spinal* | 72,068 |
| S25 | TI epidural* OR AB epidural* | 12,597 |
| S26 | TI "central neuraxial*" OR AB "central neuraxial*" | 88 |
| S27 | S19 OR S20 OR S21 OR S22 OR S23 OR S24 OR S25 OR S26 | 306,324 |
| S28 | S14 AND S18 AND S27 | 1,192 |

**Table 4. Search Results for IEEE Xplore (IEEE) [1988 – present]; Total Records 855**

| ("artificial intelligence" OR AI OR "deep learning" OR "machine learning" OR automat* OR segment* OR "computer vision" OR "machine intelligence" OR "computer aid" OR "neural network") AND (ultraso* OR sonog*) AND (anaesthe* OR anesthe* OR regional OR block* OR spinal* OR epidural OR “central neuraxial”) |
| --- |

**Table 5. Search Results for ACM Digital Library (ACM) [1951 – present]; Total Records 457**

| *12 Results for: [[Title: "artificial intelligence"] OR [Title: ai] OR [Title: "deep learning"] OR [Title: "machine learning"] OR [Title: automat*] OR [Title: segment*] OR [Title: "computer vision"] OR [Title: "machine intelligence"] OR [Title: computer aid*] OR [Title: neural network*]] AND [[Title: ultraso*] OR [Title: sonog*]] AND [[Title: anaesthe*] OR [Title: anesthe*] OR [Title: regional] OR [Title: block*] OR [Title: spinal*] OR [Title: epidural*] OR [Title: central neuraxial*]]* |
| --- |
| *445 Results for: [[Abstract: "artificial intelligence"] OR [Abstract: ai] OR [Abstract: "deep learning"] OR [Abstract: "machine learning"] OR [Abstract: automat*] OR [Abstract: segment*] OR [Abstract: "computer vision"] OR [Abstract: "machine intelligence"] OR [Abstract: computer aid*] OR [Abstract: neural network*]] AND [[Abstract: ultraso*] OR [Abstract: sonog*]] AND [[Abstract: anaesthe*] OR [Abstract: anesthe*] OR [Abstract: regional] OR [Abstract: block*] OR [Abstract: spinal*] OR [Abstract: epidural*] OR [Abstract: central neuraxial*]]* |

**Table 6. Names and URL for Databases of Non-Academic Sources of Data**

*Registries of Scoping and Systematic Reviews*

- JBI Database of Systematic Reviews and Implementation Reports

<https://csu-sfsu.primo.exlibrisgroup.com/discovery/fulldisplay?vid=01CALS_SFR:01CALS_SFR&tab=jsearch_slot&docid=alma991067665362702901&searchScope=EVERYTHING&context=L&lang=en>

- Cochrane Database of Systematic Reviews

<https://www.cochranelibrary.com/cdsr/reviews>

- International Prospective Register of Systematic Reviews (PROSPERO)

<https://www.crd.york.ac.uk/prospero>

*International Committee of Medical Journal Editors (ICMJE) approved clinical trial registries*

- Australian & New Zealand Clinical Trials Registry

[www.anzctr.org.au](http://www.anzctr.org.au)

- US National Library of Medicine online database

[www.clinicaltrials.gov](http://www.clinicaltrials.gov)

- WHO and ICJME

[www.ISRCTN.org](http://www.ISRCTN.org)

- University hospital Medical Information Network

[www.umin.ac.jp.ctr](http://www.umin.ac.jp.ctr)

- Dutch Clinical Trial Register

[www.onderzoekmetmensen.nl/en](http://www.onderzoekmetmensen.nl/en)

- European Union Drug Regulating Authorities Clinical Trials Database

<https://eudract.ema.europa.eu/>

*Other Databases*

- EU Clinical Trials Register

[www.clinicaltrialsregister.eu/](http://www.clinicaltrialsregister.eu/)

- WHO Clinical Trials Registry Platform

<http://www.who.int/ictrp/search/en/>

- Ethos

<http://ethos.bl.uk/Home.do>

- FDA MAUDE

<https://www.accessdata.fda.gov/scripts/cdrh/cfdocs/cfmaude/search.cfm>

- FDA 510k

<https://www.accessdata.fda.gov/scripts/cdrh/cfdocs/cfpmn/pmn.cfm>

- FDA Recall

<https://www.accessdata.fda.gov/scripts/cdrh/cfdocs/cfres/res.cfm>

- MHRA Product Database

<https://products.mhra.gov.uk>

*Specialist Societies*

- African Society for Regional Anesthesia

<http://afsra.org>

- American Society of Regional Anesthesia & Pain Medicine

<https://www.asra.com>

- Asian and Oceanic Society of Regional Anaesthesia and Pain Medicine

<https://aosrapm.org>

- European Society of Regional Anaesthesia and Pain Therapy

<https://esraeurope.org>

- Latin American Society of Regional Anesthesia

[www.lasra.com.br](http://www.lasra.com.br)

- Regional Anaesthesia UK

<https://www.ra-uk.org>

*Commercial Organisations*

- GE Healthcare (Chicago, IL, USA)

<https://www.gehealthcare.co.uk>

- HiCura (Singapore)

<https://hicuramedical.com>

- Intelligent Ultrasound (Cardiff, UK)

<https://www.intelligentultrasound.com>

- Mindray (Shenzhen, China)

<https://www.mindray.com>

- Rivanna Medical (Charlottesville, VA, USA)

<https://www.rivannamedical.com>

- Samsung (Suwon, South Korea)

<https://www.samsung.com>

- SmartAlpha (Ankara, Turkey)

<https://www.nerveblox.com>

**Table 7. Data Extraction Form**

| **Study ID** | |
| --- | --- |
| **Details of Publication** | |
| **Title** |  |
| **Author(s)** |  |
| **Year** |  |
| **Country of origin** |  |
| **Language of source** |  |
| **Type of source:**   - Academic (full paper/abstract/unpublished and field of publication/authors e.g., computer science, clinical) - Other literature (e.g., trials registry, industry material) |  |
| **Nature of Work** | |
| **Reported according to formal guidelines?**  (E.g., CONSORT-AI, DECIDE-AI) |  |
| **Aim of Assessment** (accuracy or utility) |  |
| **New technology or validate findings from earlier study(ies)?** |  |
| **Intervention**   - AI methodology (techniques used) - Task of AI system (e.g., bounding box, segmentation, tracking) - Training data (e.g., type/size of training dataset, including scan subjects/images & who labels data) - Comparison (e.g., to human expert) |  |
| **Population Assessed:**   - Number of subjects - Subject demographics (age, gender, BMI**)** |  |
| **Type/size of test dataset** (images/videos) |  |
| **Outcomes:**   - Accuracy - clinical (e.g., expert opinion) or non-clinical (e.g., Dice metric) - Utility |  |
| **Key Findings that relate to this Scoping Review** |  |
| **Any Additional Information** |  |
