# Supplementary material for: Artificial intelligence for ultrasound scanning in regional anaesthesia: a scoping review of the evidence from multiple disciplines
Source: Br J Anaesth. 2024 Mar 5;132(5):1049–62. doi: 10.1016/j.bja.2024.01.036 (PMC11103083; doi:10.1016/j.bja.2024.01.036)
Supplement: Multimedia component 2 [file mmc2.docx]

**Artificial Intelligence for Ultrasound Scanning in Regional Anaesthesia: A Scoping Review of the Evidence from Multiple Disciplines**

**SUPPLEMENTARY MATERIAL B – OVERVIEW OF ARTIFICIAL INTELLIGENCE**

**Authors**

James S Bowness^1,2^, David Metcalfe^3,4^, Kariem El-Boghdadly^5,6^, Neal Thurley^7^, Megan Morecroft^8^, Thomas Hartley^8^, Joanna Krawczyk^2^, J Alison Noble^10^, Helen Higham^1,11^

**Author Institutions**

1. Nuffield Department of Clinical Neurosciences, University of Oxford
2. Department of Anaesthesia, Aneurin Bevan University Health Board
3. Nuffield Department of Orthopaedics, Rheumatology & Musculoskeletal Sciences, University of Oxford
4. Emergency Medicine Research in Oxford (EMROx), Oxford University Hospitals NHS Foundation Trust
5. Department of Anaesthesia and Peri-operative Medicine, Guy’s & St Thomas’s NHS Foundation Trust
6. Centre for Human and Applied Physiological Sciences, King’s College London
7. Bodleian Health Care Libraries, University of Oxford, UK
8. Faculty of Medicine, Health & Life Sciences, University of Swansea
9. Intelligent Ultrasound
10. Institute of Biomedical Engineering, University of Oxford
11. Nuffield Department of Anaesthesia, Oxford University Hospitals NHS Foundation Trust

**Corresponding Author**

James S Bowness

OxSTaR Centre, Nuffield Division of Anaesthetics, Nuffield Department of Clinical Neurosciences, John Radcliffe Hospital, Oxford, OX3 9DU

Tel: 0044 1865 231 510. E-mail: [james.bowness@jesus.ox.ac.uk](mailto:james.bowness@jesus.ox.ac.uk)

**Overview of Artificial Intelligence, Machine Learning and Deep Learning**

The term artificial intelligence (AI) was first coined by John McCarthy in 1955^1^ and describes a field that aims to enable computers to perform tasks normally associated with human intelligence.^1-3^ Computer vision, which commonly employs AI, describes techniques that allow computers to derive meaning from the visual world.^4^ Machine learning (ML) is a set of techniques within AI which allows computers to learn (improve performance of a particular task with experience).^2^ This typically involves optimising the performance of an algorithm (a set of rules-based problem solving instructions implemented by the computer) for a given task, by exposing it to data,^2^ a process called training the algorithm. If this data is labelled with the ‘correct’ classification (ground truth), this approach is called supervised ML. In unsupervised ML, algorithms learn from unlabelled data. Deep learning (DL) is a subset of techniques within ML that use a network of artificial neurones (mathematical functions) arranged in layers, providing conceptual ‘depth’. This network is often referred to as an artificial (or convolutional) neural network (ANN or CNN). During training and subsequent deployment of an algorithm, data presented to the network is processed at each layer, to extract progressively more information before assembling this at the end of processing to produce the output.

A more detailed review of these areas is provided below and an accompanying glossary of terms is provided in Table 1. The following description is most pertinent to ultrasound-guided regional anaesthesia (UGRA) and has been expanded from our previous account in Lloyd et al (2022).^3^

**Machine Learning**

*The Rise of Machine Learning*

Machine learning, and the subfield of DL, are particularly well suited to image analysis as these are powerful techniques for identifying underlying patterns in data (in this case, pixels). The rise of DL is often attributed to the increase in the amount of data available to construct datasets, along with advances in central/graphic processing units (CPUs/GPUs) which have allowed models to be trained more efficiently. Whilst model training is often the most computationally intensive element of the DL pipeline, the ability to process and classify pixels in real (or near-real) time using trained models is made possible by powerful integrated CPUs and GPUs being part of modern devices. For example, if an algorithm is to identify sono-anatomical structures on an ultrasound image, it must determine what ‘class’ each pixel in that image falls into (segmentation). When supporting real-time ultrasound scanning, this must be done on each frame of the ultrasound feed, which refreshes at a rate of about 25 – 30 frames/second. Ultrasound images are typically 512 x 512 pixels,^5^ meaning that 262,144 pixels must be classified in each frame. Given the frame refresh rate, this means at least 6,553,600 pixels must be classified each second, or 393,216,000 each minute. It must be noted that images are often resized (e.g., to 224 x 224) and other methods (e.g., an AI frame rate <25 frames/second) are employed to improve training and inference times. Nevertheless, this description serves to illustrate the high data burden of DL techniques and why they have only become feasible on a large scale due to the progress in computational power.

*Machine Learning Categories*

As briefly described above, ML can be divided into categories based on the approach used to train the models. In supervised ML, the model is trained using labelled data (e.g., if the model was intended to predict whether an image contains a cat or a dog, images in the training data would need to be labelled as either cat or dog).^3^ The data label is commonly referred to as the ‘ground truth’, which is accepted as the outcome/answer/prediction which is known to be true or real. This approach allows direct oversight of the model training and intended output, however it is very labour-intensive to gather and label such large volumes of data. In unsupervised ML, the training data contains no labels and the model identifies areas of similarity within the data. This allows training on large volumes of data to be undertaken more quickly (as the time to label the data is not required), however it provides less direction for the initial training and trends identified in the data may not be meaningful. Semi-supervised ML uses a combination of the two approaches and can be useful when it is not possible to label all the training data (due to constraints of time or cost). A common approach is to start model training using unlabelled data, then review the output and refine it with labelled data.

**Model-Based Image Segmentation & Convolutional Neural Networks**

*Model-Based Image Segmentation*

In model-based image analysis, an algorithm is encoded that defines a mathematical function or combination of functions. These functions contain modifiable (learnable) parameters, which are initially random or set to zero. As the model is trained, these parameters are optimised to enhance the performance of the algorithm for the given task. This is a powerful technique, as training data can be selected to represent the diversity of the intended target, so the models can incorporate this variation in the prediction. Also, ultrasound imaging contains background noise (echoes of the ultrasound waveform which causes interference)^6^ – as this is present in the training data, the model can take this into account when it is deployed on previously unseen data.

*Convolutional Neural Networks*

A feature of DL is that the models commonly contain a CNN. These are formed by stacking multiple layers of functions (e.g., convolution, activation, pooling) that transform the input into a set of features. These features are then classified by the CNN, typically using a fully connected ANN. The artificial neurones within convolutional layers of the CNN are mathematical functions which receive one or more input values and produces an output which is passed on to other neurones in the network. Each input is adjusted by a modifiable weight, which allows the model to learn if the alteration is a useful feature or not (these ‘learnable’ parameters are what is updated during model training).

A section of the input data (initially an ultrasound image) is presented to the initial convolutional layer of the network, which contains multiple filters. These input data are convolved with the filters to produce an abstracted set of features called a feature map. The size of the feature map produced can be controlled by a number of parameters, such as the filter size (how much of the original image the filter looks at), the stride (how much the filter moves across the image at each step of the convolution) and padding (a resizing function to ensure that input data has the appropriate size/shape at each stage of the CNN). As the number of neurones in a layer is typically not the same as the number of input values from the initial image or subsequent image arrays/feature maps produced by layers in the network, each neurone receives a vector of values referring to specific locations in the input data to retain spatial information. The data is passed to the final layer, which outputs the model prediction (e.g., whether each pixel in an input ultrasound image is artery, bone, fascia, muscle, nerve, vein). In UGRA, a common early approach is to apply a colour to each pixel to depict which class it belongs to (e.g., red for artery) (Figure 1).

*Mathematical Underpinning of CNNs*

Linear algebra is fundamental in designing neural networks. Numerical data arranged as a matrix (an array of numbers) or vectors (a special case of matric, with a single row or column of numbers) can be efficiently stored and manipulated on modern computing hardware. The inputs and outputs to the network are therefore manually expressed as vectors or matrices, which give spatial and quantifiable context to the data. Layers in a CNN are paramaterised by weights, which are also stored as matrices. In operation, the inputs are propagated through the network by carrying out a series of matrix operations as specified by each of the layers and eventually producing the network’s output.

Calculus is crucial to optimising models, where the aim is to adjust the weights of each layer to minimise the error between the predicted and desired output, as measured for examples in the training data. Error minimisation via gradient descent involves iteratively using calculus to compute the error gradient at the current value of the weights, then upgrading the weights to reduce that error.

Probability theory is vital to CNN predictions as, for example, the output of the model can be the probability of a pixel belonging to a specific class of the data (expressed as a number from 0 to 1).

*Model Architecture*

Model architecture describes how the basic DL building blocks (e.g., a convolution) are combined. There is a vast number of different architectures available, which can contribute to parts of the CNN, though the reviewed literature in this study identified a number which were commonly used (e.g., VGG, GoogLeNet, ResNet). These are all examples of CNNs which are available in the majority of DL toolkits (e.g., PyTorch, Tensorflow) and are therefore a readily-available starting point for work in this field. In some cases, it may be desirable to change components, to optimise performance for a specific domain. One of the benefits of using a commonly available model architecture is that they often come with weights that were pretrained on much larger datasets than are commonly available to the medical AI community. For example, many are available with weights trained on the ImageNet 1K,^7^ a dataset consisting of 1,281,167 training images. Although these images may not be directly applicable to the images in medical datasets, it allows the model to start from a position where it can discriminate between key concepts (e.g., textures, shapes, colours) that may be useful when subsequently learning from the medical dataset. The concept of starting from a model trained on a large-scale (general) dataset before commencing training with another (specific/smaller) dataset is referred to as transfer learning.^8^

*U-Net*

A common structure used for CNNs in computer vision in medicine is the U-Net proposed by Ronneberger et al, 2015.^9^ This model was created specifically for biomedical images and consists of two models (most commonly CNNs such as VGG or ResNet) connected together; encoders and decoders. In an initial ‘encoding’ (down sampling/contracting) limb, each layer of the neural network processes the input data (convolution functions) and uses a ‘max pooling’ function (where the maximum value of a window on the array is taken) to produce an array/feature map of reduced size to pass on to the next layer (see Figure 2). At each layer, filters extract specific information; initially there are fewer filters, and they extract simple information (e.g., straight lines), because the greater number of data points requires greater computational power. As the array passes through the network, the number of filters increases as the array itself becomes smaller, and so the computational power required to process more filters is still feasible. Layers in the subsequent ‘decoding’ (up sampling/expanding) limb have functions which increase the size of an array (transposed convolutions), to restore the feature map to the dimensions of the input image. At each layer of the encoding limb, skip connections transfer data to the corresponding layer of the decoding limb to ensure the expanding layers place new information in the correction spatial orientation.^10, 11^ The final layer of the model is the output layer, which provides the output of the model. In the case of an AI-generated colour overlay for UGRA images, a value between 0 and 1 for the probability of each pixel of the input image belonging to a specific structure class (e.g., red for artery). It is common for software engineers to use updated models for each limb of the U-Net, to give improved performance, which keeping the encoder/decoder architecture the same. This can be referred to as changing the model used in the backbone of the U-Net.^12-15^

**Training, Testing & Validation**

*Training*

An algorithm encodes the model architecture and starting values of the learnable/modifiable parameters. This must be trained on relevant data, to allow these parameters to be modified and ensure the model can correctly predict the class of a pixel or image as its output. The CNN is exposed to the training data, the modifiable parameters are repeatedly adjusted based on the statistical correlation of examples in the training dataset and/or correlation between the training image and the label. Each time this occurs is called an epoch. With repeated exposure, the models’ parameters stabilise on the optimal values for the best overall output of the model.

This is an area of interest/concern for AI researchers as (conscious or unconscious) bias in selection of training data has a high likelihood of leading to bias in the model output. Therefore, training data must appropriately reflect the diversity of data encountered when the model will be deployed. In the context of UGRA, training data must contain examples of variant anatomy if developers expect the final model to perform as expected on these infrequent cases (‘edge cases’).

*Testing & Validation*

Progress is serially testing during training (after each epoch), by measuring the ‘loss function’ of the model. This is the difference between the model output and the ‘ground truth’ (true value). Typically, when training data is collected, it is partitioned into three:

- Training: develop statistical correlations to modify learnable parameters of the model (80%)
- Test: assess progress by calculating the loss function after each period (epoch) of training (10%)
- Holdout (internal validation): assess model performance when training is complete (10%).

Training data must not also appear in the test or holdout/validation data, as performance of the model with appear artificially enhanced. Once internal validation (assessing against holdout data) has been completed, it is important that models then undergo an external validation. In this process, model performance is compared to a ground truth for a dataset that has been acquired in a different temporal or geographical setting. This is an essential step to ensure that the models are generalisable and have not become accustomed to peculiarities that are specific to the training dataset (overfitting).**^1^**

A frustrating feature of DL is that the terms testing, holdout and validation are used in different contexts by different elements of the community.**^3^** It is therefore essential to scrutinise the meaning of ‘testing’ and ‘validation’ when interpreting results of model performance.

*Metrics for Assessment*

Another feature of this field which impedes understanding by the non-expert, and therefore hinders widespread understanding/uptake, in consistency in the metrics used to assess accuracy of AI model predictions in computer vision.

The most commonly cited indices for comparing overlap for segmentation areas are the Dice metric (Dice similarity coefficient) and intersection over union (IoU, Jaccard index). The F/F1 score is typically used for classification tasks and the Hausdorff metric for segmentation of lines rather than areas. Definitions are provided in Table 1. There is no clear evidence that one method is superior to another, however consistent use of a single metric would better enable readers/users to compare performance of one model with another.

Furthermore, assessment of models must take into account the threshold for acceptance (discriminative performance) and the frequency with which a given prediction occurs naturally (calibration). This is discussed in more detail by van der Meijden et al (2023).^1^

**“Pre-Deep Learning” (AI & Medical Computer Vision Prior to 2016/2017)**

In the papers reviewed prior from the period before DL became prevalent, there is often a pipeline employed, pre-processes an image, extracts features, and then learns to classify the image based on the features. The pre-processing typically takes the forms of de-noising or de-speckling,^16-26^ to allow for clearer features to be extracted. Once the images are processed, a number of techniques are used to extract meaningful features from the data. Examples include the Hadamard transformation,^27, 28^ Gabor filters, clustering or template matching.^29^ When features are extracted and selected, they are passed to a classifier. Examples of classifiers in the literature are support vector machines,^18, 19, 30^ simple feed-forward neural networks,^27, 31^ or Gaussian filters.^32^

An important distinction between the more recent DL approaches are the older techniques is the use of sliding windows. Modern DL techniques typically take the whole image as an input and, in the case of segmentation, classify each pixel of the input image. Prior to DL, this was typically done by a sliding window approach.^33^ With this techniques, only a small region of the image is classified at a time but, when repeated over all areas of the image, a full classification is built up. This also help with localisation tasks where certain regions of the image can be classified as containing an important structure (e.g., a nerve). However, a disadvantage to this approach is that all pixels in the window are given the relevant class when the structure itself may only occupy a small region of the window. Thus, approaches were developed to use techniques to fit classification around structures within the window (e.g., active contour modelling).^34, 35^

**References**

1 van der Meijden SL, Arbous MS, Geerts BF. Possibilities and challenges for artificial intelligence and machine learning in perioperative care. *BJA Educ* 2023; **23**: 288-94

2 Bowness J, El-Boghdadly K, Burckett-St Laurent D. Artificial intelligence for image interpretation in ultrasound-guided regional anaesthesia. *Anaesthesia* 2021; **76**: 602-7

3 Lloyd J, Morse R, Taylor A, et al. Artificial Intelligence: Innovation to Assist in the Identification of Sono-anatomy for Ultrasound-Guided Regional Anaesthesia. *Adv Exp Med Biol* 2022; **1356**: 117-40

4 Bowness JS, El-Boghdadly K, Woodworth G, Noble JA, Higham H, Burckett-St Laurent D. Exploring the utility of assistive artificial intelligence for ultrasound scanning in regional anesthesia. *Reg Anesth Pain Med* 2022; **47**: 375-9

5 Dandu RV. Storage media for computers in radiology. *Indian J Radiol Imaging* 2008; **18**: 287-9

6 Michailovich OV, Tannenbaum A. Despeckling of medical ultrasound images. *IEEE Trans Ultrason Ferroelectr Freq Control* 2006; **53**: 64-78

7 Russakovsky O, Deng J, Su H, et al. ImageNet Large Scale Visual Recognition Challenge. *International Journal of Computer Vision* 2015; **115**: 211-52

8 Tian D, Zhu B, Wang J, et al. Brachial Plexus Nerve Trunk Recognition From Ultrasound Images: A Comparative Study of Deep Learning Models. *IEEE Access* 2022; **10**: 82003-14

9 Ronneberger O, Fischer P, Brox T. U-net: Convolutional networks for biomedical image segmentation. 234-41

10 Wang Y, Geng J, Zhou C, Zhang Y. Segmentation of ultrasound brachial plexus based on U-Net. *2021 International Conference on Communications, Information System and Computer Engineering (CISCE).* 482-5

11 Ding Y, Yang Q, Wu G, Zhang J, Qin Z. Multiple instance segmentation in brachial plexus ultrasound image using BPMSegNet. *arXiv preprint arXiv:201212012* 2020

12 Jo Y, Lee D, Baek D, et al. Optimal View Detection for Ultrasound-guided Supraclavicular Block using Deep Learning Approaches. Research Square, 2023

13 Ding Y, Yang Q, Wang Y, Chen D, Qin Z, Zhang J. MallesNet: A multi-object assistance based network for brachial plexus segmentation in ultrasound images. *Med Image Anal* 2022; **80**: 102511

14 Wang R, Shen H, Zhou M. Ultrasound nerve segmentation of brachial plexus based on optimized ResU-Net. *2019 IEEE international conference on imaging systems and techniques (IST).* 2020; 1-6

15 Cao Y, Tan C, Qian W, et al. Automatic Spinal Ultrasound Image Segmentation and Deployment for Real-time Spine Volumetric Reconstruction. *2022 IEEE International Conference on Unmanned Systems (ICUS).* 922-7

16 Abraham N, Illanko K, Khan N, Androutsos D. Deep learning for semantic segmentation of brachial plexus nervesin ultrasound images using U-Net and M-Net. *2019 3rd International Conference on Imaging, Signal Processing and Communication (ICISPC).* 85-9

17 Kakade A, Dumbali J. Identification of nerve in ultrasound images using U-net architecture. *2018 International conference on communication information and Computing Technology (ICCICT).* 1-6

18 Hadjerci O, Hafiane A, Makris P, Conte D, Vieyres P, Delbos A. Nerve localization by machine learning framework with new feature selection algorithm. *International Conference on Image Analysis and Processing.* 246-56

19 Hadjerci O, Hafiane A, Conte D, Makris P, Vieyres P, Delbos A. Ultrasound median nerve localization by classification based on despeckle filtering and feature selection. *IEEE International conference on image processing (ICIP).* 4155-9

20 Hadjerci O, Hafiane A, Morette N, Novales C, Vieyres P, Delbos A. Assistive system based on nerve detection and needle navigation in ultrasound images for regional anesthesia. Expert Systems With Applications, 2016; 64 - 77

21 Hadjerci O, Hafiane A, Vieyres P, Conte D, Makris P, Delbos A. Computer-aided detection system for nerve identification using ultrasound images: A comparative study. Informatics in Medicine Unlocked, 2016; 29 - 43

22 Yu S, Tan KK, Sng BL, Li S, Sia AT. Automatic identification of needle insertion site in epidural anesthesia with a cascading classifier. *Ultrasound Med Biol* 2014; **40**: 1980-90

23 Yu S, Tan KK. Classification of lumbar ultrasound images with machine learning. *Simulated Evolution and Learning: 10th International Conference*, 2014; 287 - 98

24 Yu S, Tan KK, Sng BL, Li S, Sia AT. Lumbar Ultrasound Image Feature Extraction and Classification with Support Vector Machine. *Ultrasound Med Biol* 2015; **41**: 2677-89

25 Yu S, Tan K, Sng B, Li S, Sia A. Real-time automatic spinal level identification with ultrasound image processing. *2015 IEEE 12th International Symposium on Biomedical Imaging (ISBI).* 243-6

26 Giraldo JJ, Álvarez MA, Orozco Á A. Peripheral nerve segmentation using Nonparametric Bayesian Hierarchical Clustering. *Annu Int Conf IEEE Eng Med Biol Soc* 2015; **2015**: 3101-4

27 Pesteie M, Abolmaesumi P, Ashab HA, et al. Real-time ultrasound image classification for spine anesthesia using local directional Hadamard features. *Int J Comput Assist Radiol Surg* 2015; **10**: 901-12

28 Pesteie M, Lessoway V, Abolmaesumi P, Rohling RN. Automatic Localization of the Needle Target for Ultrasound-Guided Epidural Injections. *IEEE Trans Med Imaging* 2018; **37**: 81-92

29 Ikhsan M, Kok Kiong T, Ting Ting O, Lew JP, Ban Leong S. Gabor-based automatic spinal level identification in ultrasound. *Annu Int Conf IEEE Eng Med Biol Soc* 2017; **2017**: 3146-9

30 Yusong L, Shuang Y, Kok Kiong T, Tildsley P, Sia AT, Ban Leong S. Development of a real-time lumbar ultrasound image processing system for epidural needle entry site localization. *Annu Int Conf IEEE Eng Med Biol Soc* 2016; **2016**: 4093-6

31 Yu S, Tan KK. Classification of lumbar ultrasound images with machine learning. *Asia-Pacific conference on simulated evolution and learning.* 287-98

32 González JG, Alvarez MA, Orozco AA. Automatic segmentation of nerve structures in ultrasound images using Graph Cuts and Gaussian processes. *37th Annual International Conference of the IEEE Engineering in Medicine and Biology Society (EMBC).* 3089-92

33 Baby M, Jereesh A. Automatic nerve segmentation of ultrasound images. *2017 International Conference of Electronics, Communication and Aerospace Technology (ICECA).* 107-12

34 Hafiane A, Vieyres P, Delbos A. Phase-based probabilistic active contour for nerve detection in ultrasound images for regional anesthesia. *Comput Biol Med* 2014; **52**: 88-95

35 Akkasaligar PT, Koolagudi SG, Biradar S, Hotagi P, Badiger S. Highlighting Nerves in Images for Ultrasound Guided Regional Anesthesia. *2020 IEEE Bangalore Humanitarian Technology Conference (B-HTC).* 1-6

**Figure 1. Colour overlay generated by AI model for axillary-level brachial plexus ultrasound image. Blue = bone; orange = fascia; red = artery; yellow = nerve.**

**Figure 2. Simplified schematic of a U-Net architecture**

**Table 1. Glossary of Terms (adapted from Lloyd et al, 2022)^3^**

| **Term** | **Description** |
| --- | --- |
| Algorithm | A set of rules defined by the code, executed by the computer |
| Artificial Neurone | A mathematical function (receives input from neurones in the previous later and provides output to neurones in the next layer) |
| Artificial Neural Network (ANN) | See Convolutional Neural Network (CNN) |
| Bounding Box | Shape that encloses/surrounds a structure on the ultrasound image (typically with reqular lines/angles, rather than the smooth outline of segmentation e.g, square) |
| Convolution | A mathematical function/operation performed by neurones in the CNN |
| Convolutional Neural Network | A network with artificial neurones arranged in layers; the original input is fed in and processed (undergoes mathematical functions/convolutions) at each layer, before the resulting data is passed on to the next layer, until the final layer is reached which outputs a decision/classification from the whole network |
| Dice Similarity Coefficient  (Sørensen-Dice Coefficient, Dice Index/Metric) | A measure of similarity between two datasets, expressed by the following equation:  DSC = 2 x area of overlap/total area (area of union) |
| Edge Cases | An infrequent/outlier case (e.g., variant anatomy on the ultrasound image) |
| Epoch | One complete cycle of the CNN through the full training dataset |
| External Validation | Testing of the model on external data (collected in a different process to the training data, separated by time or space) to demonstrate generalisability of the model |
| F Score | A measure of a test’s accuracy, uses true positives/negatives and false positives/negatives to calculate other metics (e.g., sensitivity, specificity, precision, recall, positive predictive value, negative predictive value) |
| Ground Truth | Interpretation of the data which is known or accepted to be true |
| Hausdorff Metric | A measure of how far two datasets are from each other (calculate the distance between each datum in dataset A and each datum in dataset B, calculate the minimum distance between each point in set A and all points in set B, Hausdorff is the maximum value of these minimum distances) |
| Holdout Data | A section of the original dataset gathered, which is partitioned from the training section, to test internal validity of the model after training is complete |
| Internal Validation (Testing) | Testing of the model on holdout data (these data are collected in the same process as the training data) to satisfy developers that model training is complete |
| Intersection over Union  (Jaccard Similarity Coefficient) | Similar to Dice Similarity Co-efficient, but is simply:  IoU = area of overlap/total area (area of union) |
| Loss Function | A method to assess model performance, by measuring the difference between the model output and the ground truth (a higher loss function equates to poorer performance) |
| Parameter (of a model) | Numerical value in the model, which contributes to data processing in the artificial neuron, that can be modified during train (to alter the artificial neuron/model output) |
| Segmentation | Dividing up an image into parts which contain pixels belonging to the same class (e.g., marking the boundary of the artery) |
